# Hemorrhagic shock primes for lung vascular endothelial cell pyroptosis: role in pulmonary inflammation following LPS

**DOI:** 10.1038/cddis.2016.274

**Published:** 2016-09-08

**Authors:** Jie Yang, Yanfeng Zhao, Peng Zhang, Yuehua Li, Yong Yang, Yang Yang, Junjie Zhu, Xiao Song, Gening Jiang, Jie Fan

**Affiliations:** 1 Department of Thoracic Surgery, Shanghai Pulmonary Hospital, Tongji University School of Medicine, Shanghai 200433, China; 2 Department of Surgery, University of Pittsburgh School of Medicine, Pittsburgh, PA 15213, USA; 3 Research and Development, Veterans Affairs Pittsburgh Healthcare System, Pittsburgh, PA 15240, USA; 4 McGowan Institute for Regenerative Medicine, University of Pittsburgh, Pittsburgh, PA 15219, USA

## Abstract

Hemorrhagic shock (HS) often renders patients more susceptible to lung injury by priming for an exaggerated response to a second infectious stimulus. Acute lung injury (ALI) is a major component of multiple organ dysfunction syndrome following HS and regularly serves as a major cause of patient mortality. The lung vascular endothelium is an active organ that has a central role in the development of ALI through synthesizing and releasing of a number of inflammatory mediators. Cell pyroptosis is a caspase-1-dependent regulated cell death, which features rapid plasma membrane rupture and release of proinflammatory intracellular contents. In this study, we demonstrated an important role of HS in priming for LPS-induced lung endothelial cell (EC) pyroptosis. We showed that LPS through TLR4 activates Nlrp3 (NACHT, LRR, and PYD domains containing protein 3) inflammasome in mouse lung vascular EC, and subsequently induces caspase-1 activation. However, HS induced release of high-mobility group box 1 (HMGB1), which acting through the receptor for advanced glycation end products initiates EC endocytosis of HMGB1, and subsequently triggers a cascade of molecular events, including cathepsin B release from ruptured lysosomes followed by pyroptosome formation and caspase-1 activation. These HS-induced events enhance LPS-induced EC pyroptosis. We further showed that lung vascular EC pyroptosis significantly exaggerates lung inflammation and injury. The present study explores a novel mechanism underlying HS-primed ALI and thus presents a potential therapeutic target for post-HS ALI.

Hemorrhagic shock (HS) often renders patients more susceptible to a secondary stimulus (e.g., infection) resulting in the development of systemic inflammatory response syndrome (SIRS) and multiple organ dysfunction syndrome (MODS) by activating and priming the inflammatory process. The underlying mechanism of how HS leads to SIRS and MODS has yet to be fully determined. Acute lung injury (ALI) is a major component of MODS and often serves as a direct cause of patient mortality.^1, 2^ The lung vascular endothelium is an active organ that critically contributes to the pathogenesis of ALI following trauma, sepsis, and shock by affecting pulmonary and systemic homeostasis, including secretion of cytokines, chemokines, and adhesion molecules.^3, 4^ There is a significant gap in our knowledge concerning the mechanisms of HS regulation of lung endothelial cell (EC) activation and death, and subsequent promotion of lung inflammation.

Pyroptosis is a caspase-1-dependent form of regulated cell death that is stimulated by a range of microbial infections and non-infectious stimuli.^5, 6^ Morphologically, pyroptosis is characterized by plasma membrane rupture, which results in the release of intracellular contents,^7, 8, 9, 10, 11^ and cleavage of chromosomal DNA.^7, 11, 12, 13, 14^

The magnitude of caspase-1 activation is important for the fate of cells. Low level of activation of caspase-1 might be necessary for cell survival in response to external stimulations.^15^ However, over-activation of caspase-1 may serve as a premise for cell pyroptosis.^5, 6^ The platform for caspase-1 activation includes inflammasome and pyroptosome. The former comprises of NOD-like receptors (NLRs) or AIM2 receptor, caspase-1, and apoptosis-associated speck-like protein containing a CARD domain (ASC); and the latter is composed of oligomerized ASC dimers.^16^ We have previously reported that HMGB1 has a critical role in activation of inflammasome and pyroptosome in macrophages in a setting of HS.^17, 18^

In this study, we demonstrated an important role of HS in priming for LPS-induced lung EC pyroptosis. We showed that LPS through TLR4 activates Nlrp3 inflammasome in mouse lung vascular EC (MLVEC), and subsequently induces caspase-1 activation. However, HS induced release of high-mobility group box 1 (HMGB1), which acting through the receptor for advanced glycation end products (RAGE) initiates EC endocytosis of HMGB1, and subsequently triggers a cascade of molecular events, including cathepsin B (CatB) release from ruptured lysosomes followed by pyroptosome formation and caspase-1 activation. These HS-induced events enhance LPS-induced EC pyroptosis. We further showed that lung vascular EC pyroptosis significantly exaggerates lung inflammation and injury. The present study explores a novel mechanism underlying HS-primed ALI and thus presents a potential therapeutic target for ALI induced after HS.

## Results

### HS primes for lung endothelial cell pyroptosis in response to LPS through HMGB1-RAGE signaling

Pyroptosis is characterized by caspase-1 activation and DNA fragmentation. To determine a priming role for HS in enhancing lung EC pyroptosis in response to LPS, we used a ‘two-hit' mouse model sequentially treated with HS and LPS i.t. (intratracheally) (HS–LPS), as described in Materials and Methods. Lung tissue was harvested at 24 h after LPS i.t., stained with Alexa Fluor 488-labeled caspase-1 FLICA, Cell Death Reagent-TMR, E-selectin, and Hoechst, and observed using confocal microscopy.

**Figure 1:**
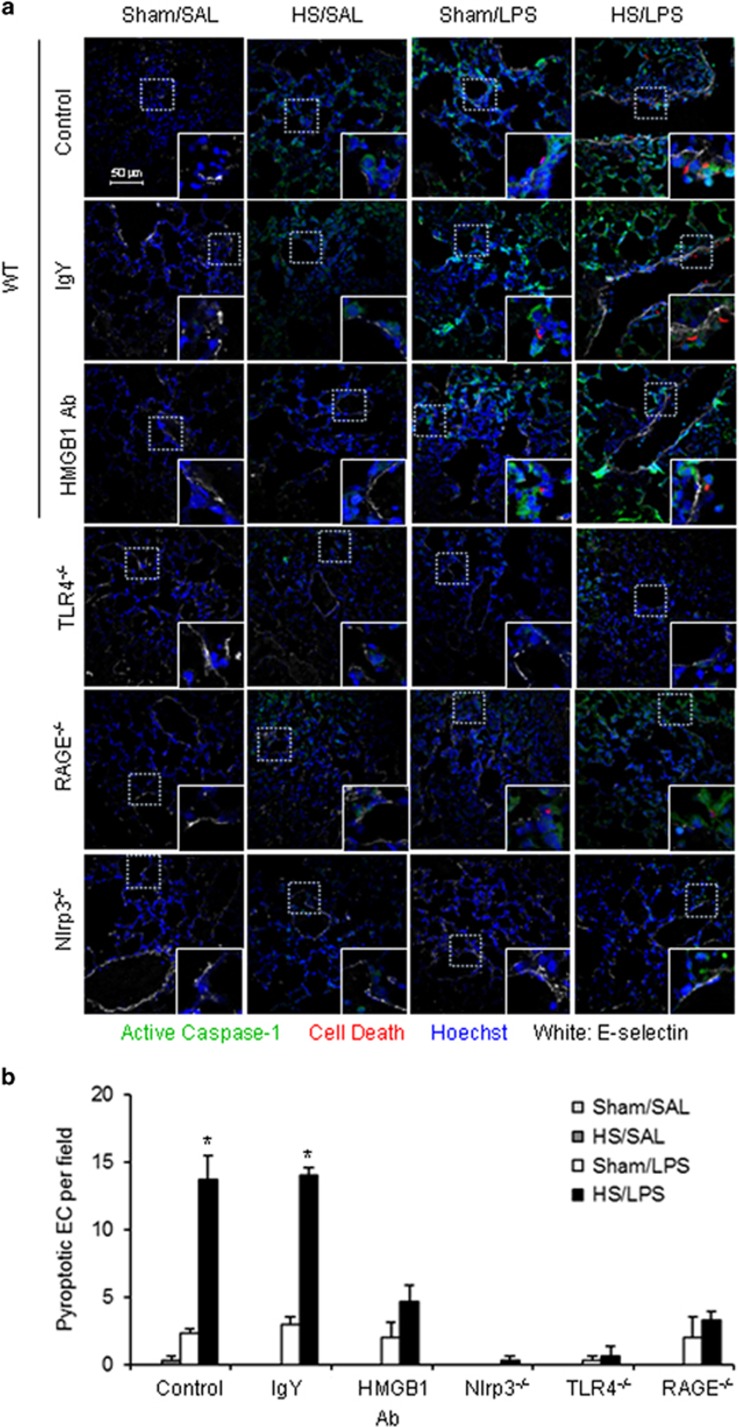
HS primes for lung endothelial cell pyroptosis in response to LPS through HMGB1-RAGE signaling. (**a**) WT (C57BL/6) mice, TLR4^−/−^ mice, RAGE^−/−^ mice, and Nlrp3^−/−^ mice were subjected to HS (HS) or sham operation (Sham) followed by LPS or saline (SAL) i.t. at 2 h after resuscitation (*n*=6 per group). Some WT mice received anti-HMGB1 Ab (2 mg/kg BW) by i.p. injection 30 min before HS or sham operation (*n*=6 per group). Lung tissue were harvested 24 h after LPS or SAL i.t. and the histological slides were stained with Cell Death Reagent-TMR (red), Alexa Fluor 488-labeled caspase-1 FLICA (green), E-selectin (white), and Hoechst (blue). Fluorescent images were obtained by confocal microscopy (original magnification × 600, higher magnification images for the selected area are shown in the respective lower right insets). Quadruple-stained cells were considered positive for pyroptotic EC. (**b**) The average number of pyroptotic EC of five random fields was counted for analysis. Data are presented as mean and S.E.M. *P<0.05 compared with the groups labeled with no asterisk

As shown in Figures 1a and b, LPS induced lung EC pyroptosis at a low level in sham animals. By contrast, the antecedent HS significantly increased EC pyroptosis in response to LPS in the HS–LPS group. In HS-SAL group, EC pyroptosis did not occur, although caspase-1 activation was detected.

Our previous studies have shown that HS causes a significant increase of HMGB1 in the serum, lung, and liver at 2 h after HS.^19^ To determine whether extracellular HMGB1 is responsible for the HS-primed lung EC pyroptosis in response to LPS, we administered neutralizing Ab against HMGB1 to mice (2 mg/kg BW) 30 min before HS. As shown in Figures 1a and b, treatment with HMGB1 Ab significantly decreased HS-primed lung EC pyroptosis as compared with the animals treated with nonspecific IgY.

In order to specify the receptors that mediate the effects of HMGB1 and LPS, we subjected TLR4^−/−^ and RAGE^−/−^ mice to the HS–LPS model. As expected, in TLR4^−/−^ mice, LPS failed to induce EC caspase-1 activation and pyroptosis (Figures 1a and b). In RAGE^−/−^ mice, LPS was still able to induce caspase-1 activation and pyroptosis in sham animals, however, RAGE deficiency suppressed the HS-enhanced pyroptosis in HS–LPS group (Figures 1a and b). Furthermore, LPS-induced caspase-1 activation and pyroptosis were prevented by Nlrp3 deficiency in either sham or HS group.

Altogether, these data suggest that LPS-induced lung EC pyroptosis is TLR4 and Nlrp3 dependent, whereas, HS-enhanced EC pyroptosis acts through HMGB1-RAGE signaling.

### *In vitro* confirmation of the role for HMGB1 in priming for lung EC pyroptosis

**Figure 2:**
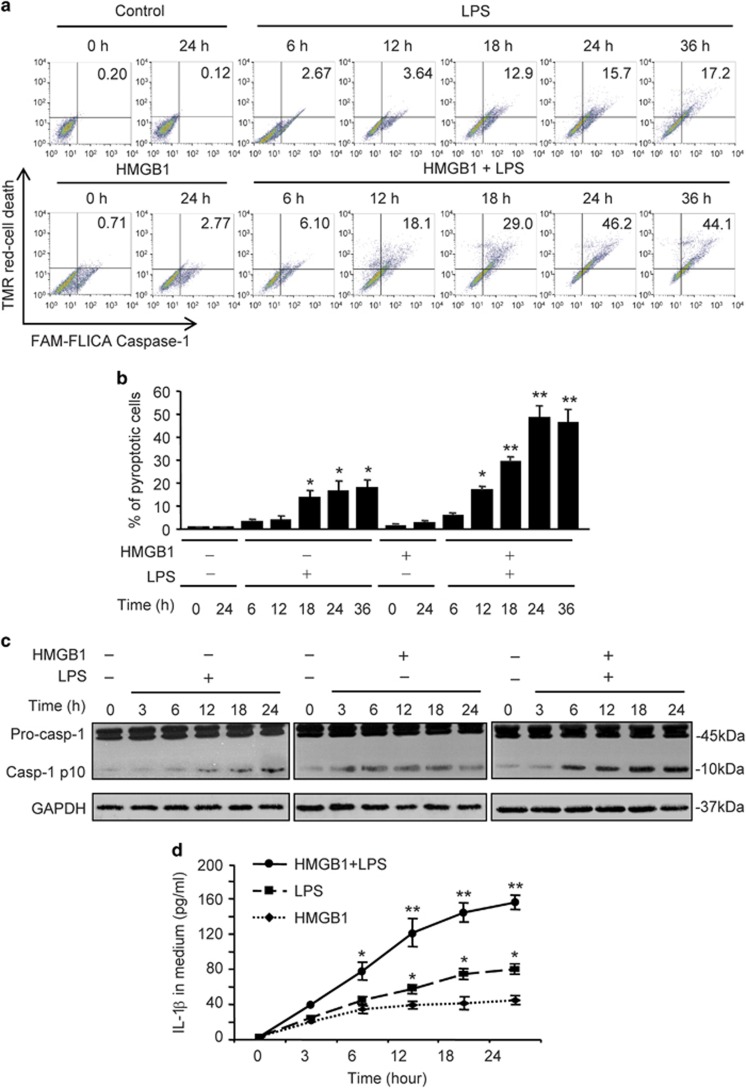

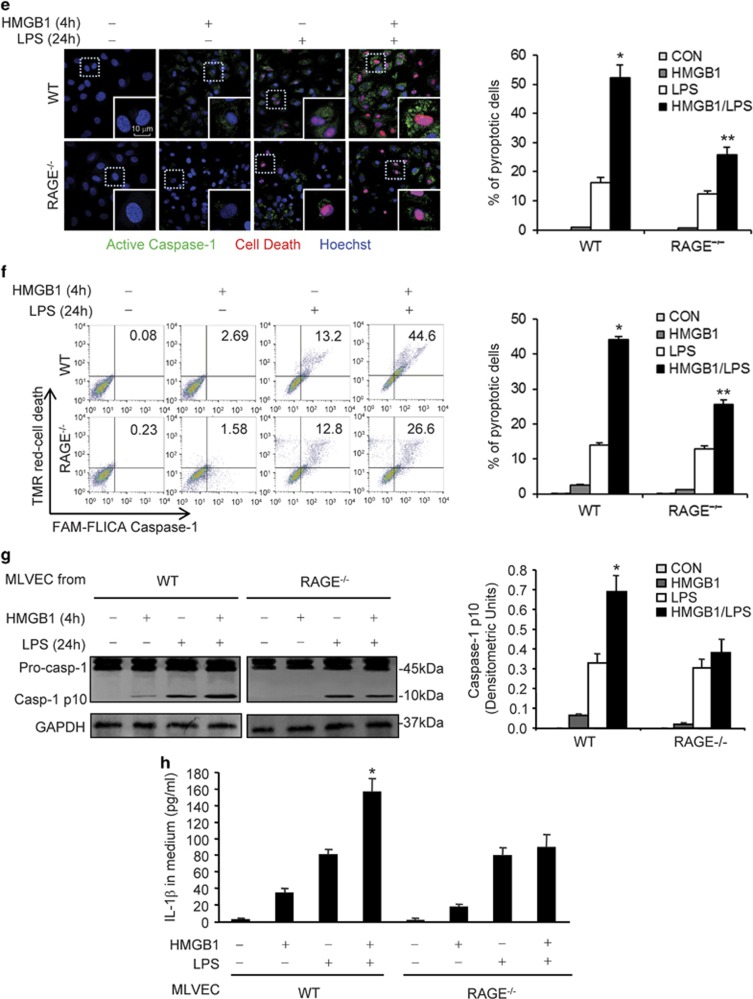
HMGB1 primes for lung EC pyroptosis in response to LPS. (**a**–**d**) MLVEC isolated from WT mice were sequentially treated with HMGB1 (0.5 *μ*g/ml) for 4 h and then with LPS (1 *μ*g/ml) for up to 36 h. Some MLVEC were treated with HMGB1 or LPS alone. MLVEC with no treatment were as control. Cells were stained with Cell Death Reagent-TMR and Alexa Fluor 488-labeled caspase-1 FLICA, and the double-stained pyroptotic cells were detected by flow cytometry (**a** and **b**). Activation of caspase-1 in cell lysate was detected by western blot (**c**). IL-1*β* in medium was measured by ELISA (**d**). (**e**–**h**) MLVEC from WT mice and RAGE^−/−^ mice were sequentially treated with HMGB1 (0.5 *μ*g/ml) for 4 h and then with LPS (1 *μ*g/ml) for 24 h. After the treatment, cells were stained with Cell Death Reagent-TMR and Alexa Fluor 488-labeled caspase-1 FLICA, the double-stained pyroptotic cells were detected by confocal microscopy (**e**) and flow cytometry (**f**). Activation of caspase-1 in cell lysate was detected by western blot (**g**). IL-1*β* in medium was measured by ELISA (**h**). All images are representatives of five independent experiments, and graphs depict the value of mean and S.E.M. *P<0.05 compared with the groups labeled with no asterisk, **P<0.05 compared with the groups labeled with no or different asterisk

To confirm the role of HMGB1 in priming for lung EC pyroptosis, we treated MLVEC with HMGB1 (0.5 *μ*g/ml) for 4 h followed by adding LPS (1 *μ*g/ml) and incubating the cells for up to 36 h. In some groups MLVEC were treated with HMGB1 or LPS alone. After the treatments, cells were stained with TMR-Cell Death Reagent and Alexa Fluor 488-labeled caspase-1 FLICA, and the double-stained pyroptotic cells were detected by flow cytometry. As shown in Figures 2a and b, HMGB1 pretreatment significantly accelerated and increased LPS-induced EC pyroptosis as compared with LPS-alone group. The alterations in intracellular caspase-1 activation, which were assessed by detecting caspase-1 cleavage product p10 fragments using western blotting, showed that HMGB1 enhanced caspase-1 cleavage in response to LPS (Figure 2c). Figure 2d shows that LPS increased IL-1*β* in the cell culture media and pretreatment with HMGB1 augmented the increase in the LPS-induced IL-1*β* release.

To determine the role of RAGE in mediating the priming effect of HMGB1 on MLVEC pyroptosis, MLVEC isolated from WT and RAGE^−/−^ mice were sequentially treated with HMGB1 (0.5 *μ*g/ml) for 4 h and then with LPS (1 *μ*g/ml) for 24 h. The MLVEC were then stained with TMR-Cell Death Reagent and Alexa Fluor 488-labeled caspase-1 FLICA, and detected by confocal microscopy and flow cytometry, respectively. RAGE deficiency significantly attenuated HMGB1-primed MLVEC pyroptosis in response to LPS, although RAGE^−/−^ did not affect LPS-induced MLVEC pyroptosis (Figures 2e and f). Likewise, RAGE deficiency failed to prevent LPS-induced caspase-1 cleavage and IL-1*β* release, whereas, significantly diminished HMGB1-primed increase in caspase-1 cleavage and IL-1*β* release in MLVEC response to LPS (Figures 2g and h).

### HMGB1 mediates HS-primed activation of Nlrp3 inflammasome in lung EC through ROS-TXNIP signaling

**Figure 3:**
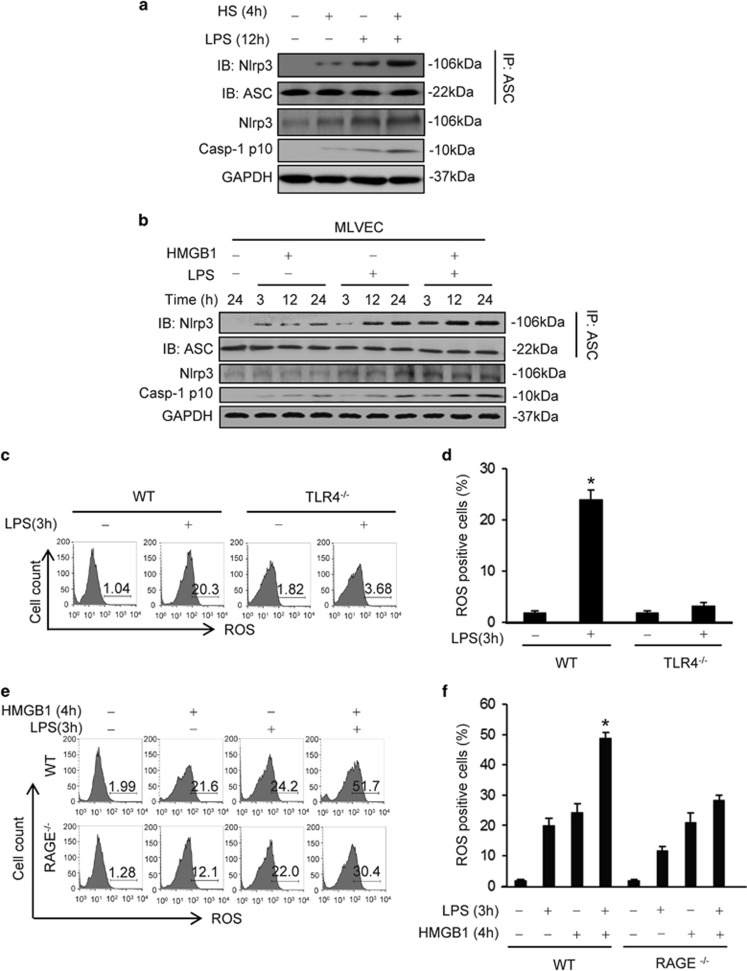

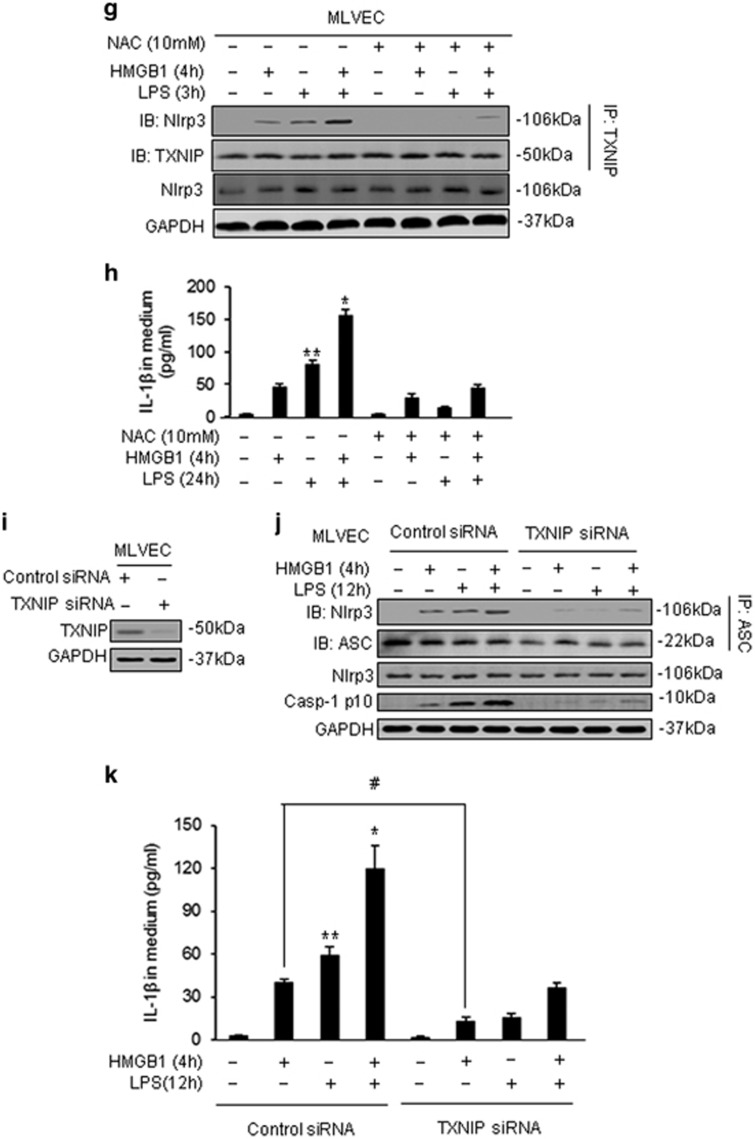
HS augments Nlrp3 inflammasome activation in lung EC through ROS-TXNIP signaling. (**a**) WT mice were subjected to HS (HS) or sham operation (Sham) followed by LPS (1 *μ*g/ml) or SAL i.t. at 2 h after resuscitation. Lung tissues were recovered 12 h after LPS or SAL i.t. The association of Nlrp3-ASC was detected using immunoprecipitation with anti-ASC antibody and immunoblotting with anti-ASC and Nlrp3 antibody. The total Nlrp3 protein expression and caspase-1 p10 fragment in the lung tissue was detected by western blot. (**b**) WT MLVEC were sequentially treated with HMGB1 (0.5 *μ*g/ml) for 4 h and then with LPS (1 *μ*g/ml) for 3, 12, and 24 h. Nlrp3-ASC association, total Nlrp3, and caspase-1 p10 fragments were detected as described in (**a**). (**c**–**f**) ROS production in live MLVEC. MLVEC from WT mice, TLR4^−/−^ mice, and RAGE^−/−^ mice were sequentially treated with HMGB1 (0.5 *μ*g/ml) for 4 h and then with LPS (1 *μ*g/ml) for 3 h. MLVEC were stained with the cell-permeable ROS detection reagent H2DFFDA (10 mM; Invitrogen Molecular Probes, Carlsbad, CA, USA) for 10 min and ROS production was then detected by flow cytometry. (**g** and **h**) WT MLVEC were sequentially treated with HMGB1 (0.5 *μ*g/ml) for 4 h and then with LPS (1 *μ*g/ml) for 3 h (**g**) and 24 h (H). For some experiments, NAC (10 mM) was added 30 min ahead of the treatment. Nlrp3 and TXNIP association was detected using immunoprecipitation and immunoblotting. IL-1*β* in medium was measured by ELISA. (**i**) TXNIP in MLVEC was knocked down using siRNA techniques as described in the Materials and Methods. At 48 h after transfection of TXNIP siRNA into MLVEC, the TXNIP protein significantly decreased as compared with control. (**j** and **k**) MLVEC were transfected with TXNIP siRNA at 48 h before treatment with HMGB1 (0.5 *μ*g/ml) for 4 h and then with LPS (1 *μ*g/ml) for 12 h. Nlrp3-ASC association, total Nlrp3, and caspase-1 cleavage in the EC were then detected using western blot (**j**) and IL-1*β* in the medium was measured by ELISA (**k**). All images are representatives of five independent experiments, and graphs depict the value of mean and S.E.M. *P<0.05 compared with the groups labeled with no or different asterisk, **P<0.05 compared with the groups labeled with no or different asterisk, ^#^*P*<0.05 between the two groups

To determine whether augmented activation of Nlrp3 inflammasome contributes to HS-primed caspase-1 activation, we examined Nlrp3 inflammasome activation in the lung by detecting the association of Nlrp3 and ASC, as well as caspase-1 fragment p10 in mouse lung tissue following the ‘two-hit' treatments of HS–LPS. Lung tissue was recovered at 12 h after LPS and the association of Nlrp3 and ASC was determined by using coimmunoprecipitation and immunoblotting. Administration of LPS to sham animals induced an increase in the association between Nlrp3 and ASC and cleavage of caspase-1 in the lung by 12 h (Figure 3a). Animals subjected to HS before LPS exhibited a noticeable increase in the association between Nlrp3 and ASC and cleavage of caspase-1 as compared with that in the lungs from sham/LPS group (Figure 3a).

We further sequentially treated WT MLVEC *in vitro* with HMGB1 for 4 h followed by LPS for up to 24 h, and detected Nlrp3-ASC association and caspase-1 cleavage. As shown in Figure 3b, MLVEC treated with HMGB1-LPS exhibited a markedly augmented association of Nlrp3 and ASC and increase in caspase-1 cleavage as compared with those in the groups treated with LPS alone (Figure 3b).

We have previously reported that reactive oxygen species (ROS)-thioredoxin-interacting protein (TXNIP) signaling mediates Nlrp3 inflammasome activation in lung EC.^20^ To determine the role of ROS-TXNIP signaling in HMGB1-primed inflammasome assembly in response to LPS, MLVEC from WT, TLR4^−/−^, and RAGE^−/−^ mice were stimulated with HMGB1 for 4 h and then with LPS for 3 h, and ROS production was detected by H2DFFDA (Invitrogen Molecular Probes, Carlsbad, CA, USA) using flow cytometry. The results showed that LPS induced a significant increase in ROS production in WT MLVEC; whereas, TLR4 deficiency markedly decreased ROS production in response to LPS (Figures 3c and d). HMGB1 pretreatment followed by LPS stimulation led to an amplified ROS production in the EC as compared with the group treated with LPS alone. RAGE deficiency, however, did not affect LPS induced EC ROS production, but significantly attenuated the ROS production induced by HMGB1-LPS treatments (Figures 3e and f). These results suggest that HMGB1 acting mainly through RAGE synergistically increases ROS production in MLVEC in response to LPS.

Furthermore, we determined the role of ROS in promoting Nlrp3-TXNIP association in MLVEC. As shown in Figure 3g, HMGB1 markedly increased the association of Nlrp3 and TXNIP in response to LPS at 3 h; while, pretreatment with NAC, a ROS scavenger, significantly attenuated the association. NAC also significantly decreased the concentrations of IL-1*β* in medium induced by HMGB1 and/or LPS at 24 h (Figure 3h).

In order to address whether the upregulated association between TXNIP and Nlrp3 was responsible for the augmented Nlrp3 inflammasome activation, we silenced TXNIP expression in MLVEC by siRNA to TXNIP. At 48 h after transfection of TXNIP siRNA into MLVEC, TXNIP protein content was significantly decreased in the EC (Figure 3i). Knockdown of TXNIP significantly reduced the HMGB1-LPS-induced association of Nlrp3 and ASC as well as caspase-1 cleavage at 12 h (Figure 3j). Consistently, TXNIP silencing decreased IL-1*β* release from the MLVEC in response to HMGB1 and/or LPS (Figure 3k).

Taken together, these data support the hypothesis that HS augments LPS-induced Nlrp3 inflammasome activation in lung EC through ROS-TXNIP signaling.

### HMGB1 endocytosis induces pyroptosome formation in lung EC

**Figure 4:**
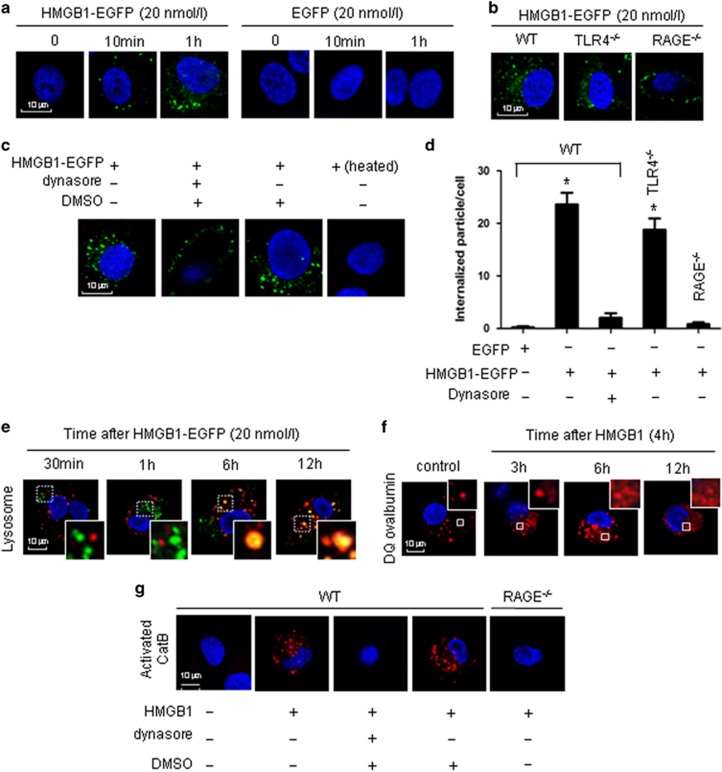
Lung EC endocytosis of HMGB1 induces lysosomal destabilization and CatB activation. (**a**) Confocal microscopy of MLVEC that were isolated from WT mice and incubated with recombinant HMGB1-EGFP (20 nmol/l) or EGFP for 0, 10 min, and 1 h. (**b**) Confocal microscopy of MLVEC that were isolated from WT, TLR4^−/−^, or RAGE^−/−^ mice and incubated with HMGB1-EGFP (20 nmol/l) for 1 h. (**c**) Confocal microscopy of WT MLVEC that were incubated with HMGB1-EGFP in the presence or absence of dynasore (30 *μ*g/ml) or DMSO (0.3%) for 1 h. WT MLVEC were also treated with heated HMGB1-EGFP (100 °C, 5 min) for 1 h. (**d**) Numerical summary of the findings from panels (**a** to **c**). Graph depicts the number of intracellular EGFP-tagged protein particles in MLVEC, which were calculated using confocal microscopy program. Mean±S.E.M, *n*=5. *P<0.01 compared with the groups labeled with no asterisk. (**e**) WT MLVEC were incubated with HMGB1-EGFP (green) and LysoTracker Red lysosome dye (red) for 30 min, 1 h, 6 h, or 12 h. Co-localization of HMGB1 and lysosome was detected using confocal microscopy. (**f**) MLVEC were treated with HMGB1 (0.5 *μ*g/ml) for 3, 6, and 12 h followed by incubation with DQ ovalbumin (red) for 1 h to visualize lysosome integrity using confocal microscopy. (**g**) MLVEC isolated from WT or RAGE^−/−^ mice were incubated with HMGB1 (0.5 *μ*g/ml) for 12 h in the absence or presence of dynasore (30 *μ*g/ml) or DMSO (0.3%). The cells were then stained with Magic Red CatB detection reagent (red) to visualize activated CatB under confocal microscopy. All images are representative of five independent experiments. Higher magnification images for the selected area are shown in the boxed insets (original magnification × 600)

We recently found in macrophages that endocytosis of HMGB1 initiates pyroptosome formation and subsequent macrophage pyroptosis.^18^ Whether this mechanism seen in myeloid cells is also valid in EC has yet to be addressed. To answer this question, we treated MLVEC with HMGB1 tagged with enhanced green fluorescent protein (HMGB1-EGFP, 20 nmol/l) and observed the cells under confocal microscope. Figure 4a shows that MLVEC internalization of HMGB1-EGFP occurred as early as 10 min after the treatment. This MLVEC internalization of HMGB1-EGFP was specific via HMGB1 and a RAGE-dependent event, as EGFP alone was not internalized, and RAGE^−/−^ prevented the HMGB1-EGFP internalization (Figure 4b). Genetic deletion of TLR4 did not block HMGB1 internalization (Figure 4b). These observations indicate that HMGB1 internalization is RAGE dependent, but TLR4 independent.

Furthermore, we found that treatment of the cells with dynamin inhibitor dynasore (30 *μ*g/ml, Sigma-Aldrich, St. Louis, MO, USA) effectively blocked HMGB1 internalization, and the dynasore solvent DMSO alone did not suppress HMGB1 endocytosis (Figure 4c). In order to address if the HMGB1-EGFP endocytosis is possibly mediated by contaminated LPS, HMGB1-EGFP was heated at 100 °C for 5 min before addition to the MLVEC. The results showed that heated HMGB1-EGFP failed to induce its endocytosis (Figure 4c). Figure 4d shows a numerical summary of the findings from Figures 4a–c. Taken together, HMGB1 acting through RAGE initiates its endocytosis into lung EC via a dynamin-dependent pathway.

In our previous study on macrophages we demonstrated a translocation of internalized HMGB1 into lysosome, which in turn, induced lysosome rupture and CatB activation. To determine if this intracellular consequence also occurs in EC, we visualized lysosomes with LysoTracker probe, a lysosome detector. We observed that HMGB1 localized in lysosomes at 6 h, and it remained there for at least 12 h (Figure 4e). We then applied fluorescence-tagged DQ ovalbumin to monitor the integrity of the lysosome compartments in the MLVEC after HMGB1 endocytosis. The fluorescence of the fluorophore BODIPY-FL (8-chloromethyl-4,4-difluoro-1,3,5,7-tetramethyl-4-bora-3a,4a-diaza-s-indacene) on DQ ovalbumin is normally quenched unless the protein is proteolytically processed into peptides in endo-lysosomal compartments.^21^ As shown in Figure 4f, in untreated control cells, processed DQ ovalbumin was localized to small vesicular and tubular lysosomes, as expected. However, notably, there were large swollen lysosomes in the EC starting from 6 h after HMGB1 treatment. The cells demonstrated a cytosolic pattern of fluorescently processed DQ ovalbumin, which suggested lysosomal rupture or leakage of lysosomal contents into the cytosol^22, 23^ in HMGB1-treated MLVEC (Figure 4f).

To address whether the observed lysosome rupture leads to CatB activation, we measured CatB activity in the cytosol using Magic Red CatB detection reagent. In WT MLVEC, a cytosolic pattern of activated CatB was observed at 12 h after HMGB1 pretreatment (Figure 4g). In contrast, HMGB1 failed to induce CatB activation in RAGE^−/−^ MLVEC as well as WT MLVEC pretreated with dynasore (Figure 4g). No effect from dynasore solvent DMSO was found in the experiments as shown in Figure 4g.

**Figure 5:**
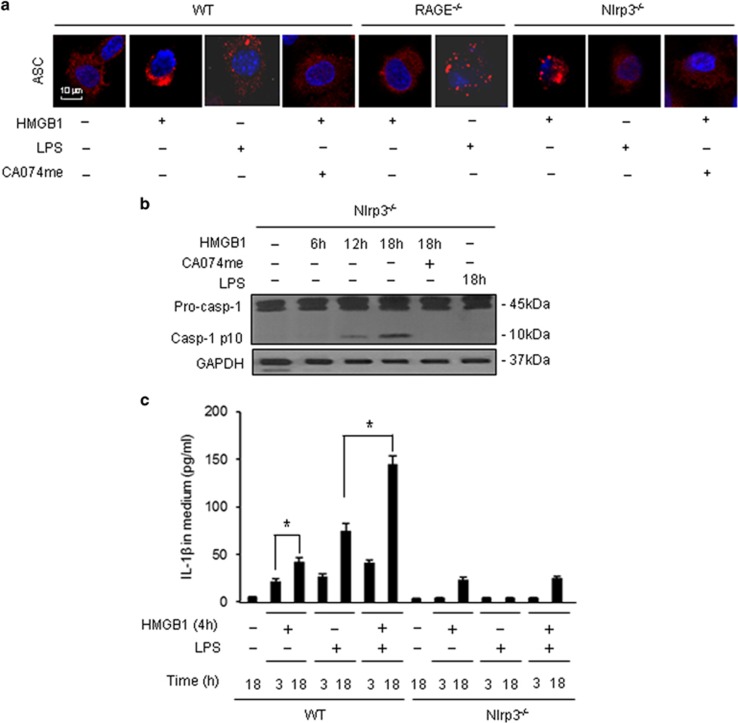
HMGB1-induced CatB activation results in pyroptosome formation and caspase-1 activation. (**a**) Confocal microscopy of immunofluorescence of ASC foci in MLVEC. MLVEC isolated from WT mice, Nlrp3^−/−^ mice, and RAGE^−/−^ mice were treated with HMGB1 (0.5 *μ*g/ml) or LPS (1 μg/ml) in the presence or absence of CA074me (10 *μ*mol/l) for 18 h, and then ASC (red) were detected by immunofluorescence and confocal microscopy. Original magnification × 600. (**b**) Nlrp3^−/−^ MLVEC were treated with HMGB1 (0.5 *μ*g/ml) in the presence or absence of CA074me (10 *μ*mol/l) for 6, 12, or 18 h. In one group, Nlrp3^−/−^ MLVEC were treated with LPS alone for 18 h. Caspase-1 activation was detected by western blot. (**c**) WT and Nlrp3^−/−^ MLVEC were sequentially treated with HMGB1 (0.5 *μ*g/ml) for 4 h and then with LPS (1 *μ*g/ml) for 3 and 18 h. IL-1*β* was detected by ELISA. All images are representatives of five independent experiments, and graphs depict the value of mean and S.E.M. *P<0.05 between the two groups

Pyroptosome, which is a supramolecular assembly of ASC dimers, recruits and maturates caspase-1 through proteolysis of pro-caspase-1 proteins.^6, 24^ To detect pyroptosome formation in MLVEC after the treatments of HMGB1 and/or LPS, we visualized the ASC foci with florescence-tagged ASC antibody and confocal microscopy. ASC foci were observed at 18 h after HMGB1 treatment in WT and Nlrp3^−/−^ MLVEC (Figure 5a). HMGB1 failed to induce ASC foci in RAGE^−/−^ MLVEC and in WT or Nlrp3^−/−^ MLVEC treated with CatB inhibitor CA074me (10 *μ*mol/l) (Figure 5a). In addition, LPS alone induced ASC foci in WT MLVEC, but not in Nlrp3^−/−^ EC, suggesting an Nlrp3-dependent formation of ASC assembly (Figure 5a). These results suggest that HMGB1 induced Nlrp3-independent ASC assembly, the pyroptosome formation, whereas, LPS induced Nlrp3-dependent ASC assembly, the Nlrp3 inflammasome formation.

Figures 5b and c show that Nlrp3 deficiency did not prevent the HMGB1-induced caspase-1 cleavage and IL-1*β* release in the MLVEC, but suppressed LPS-induced caspase-1 activation and IL-1*β* release. The data are consistent with the changes in ASC foci formation, further suggesting that HMGB1 induces pyroptosome formation, whereas, LPS induces Nlrp3 inflammasome activation. Collectively, these results show that MLVEC endocytosis of HMGB1 leads to lysosome rupture, followed by pyroptosome assembly and subsequent caspase-1 activation.

### HS-primed lung EC pyroptosis contributes to acute lung injury

**Figure 6:**
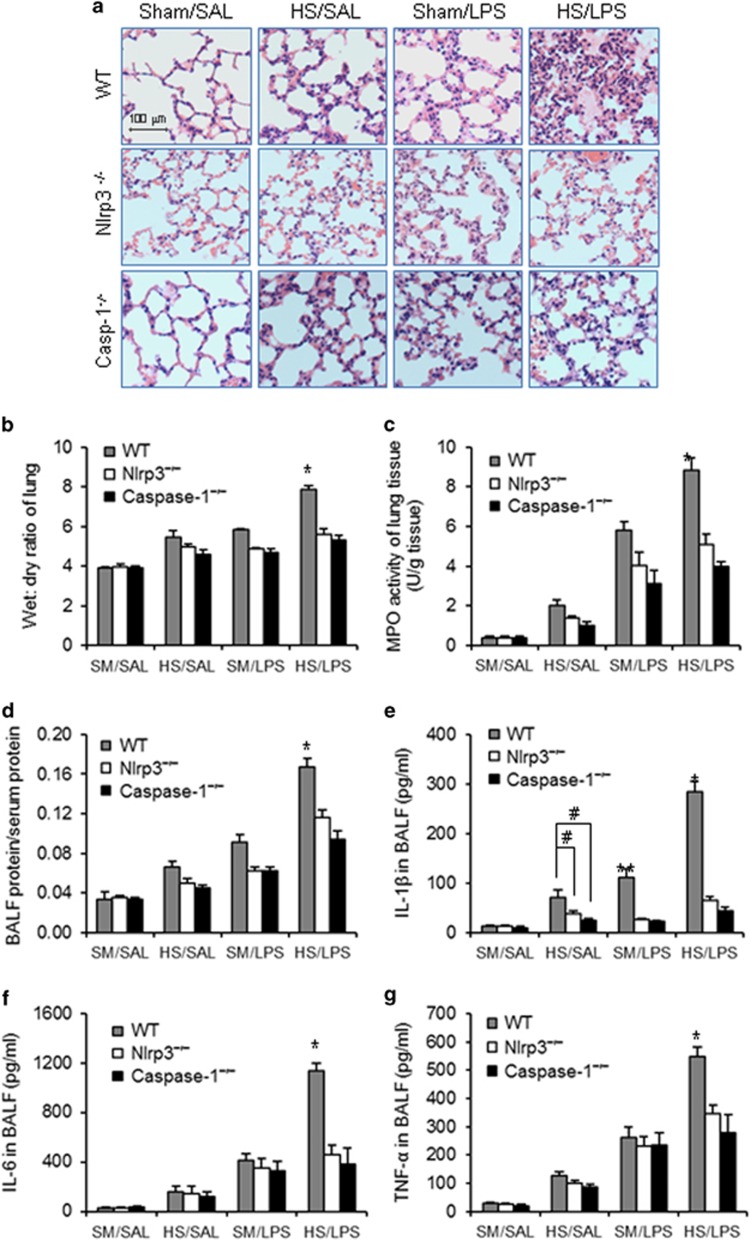

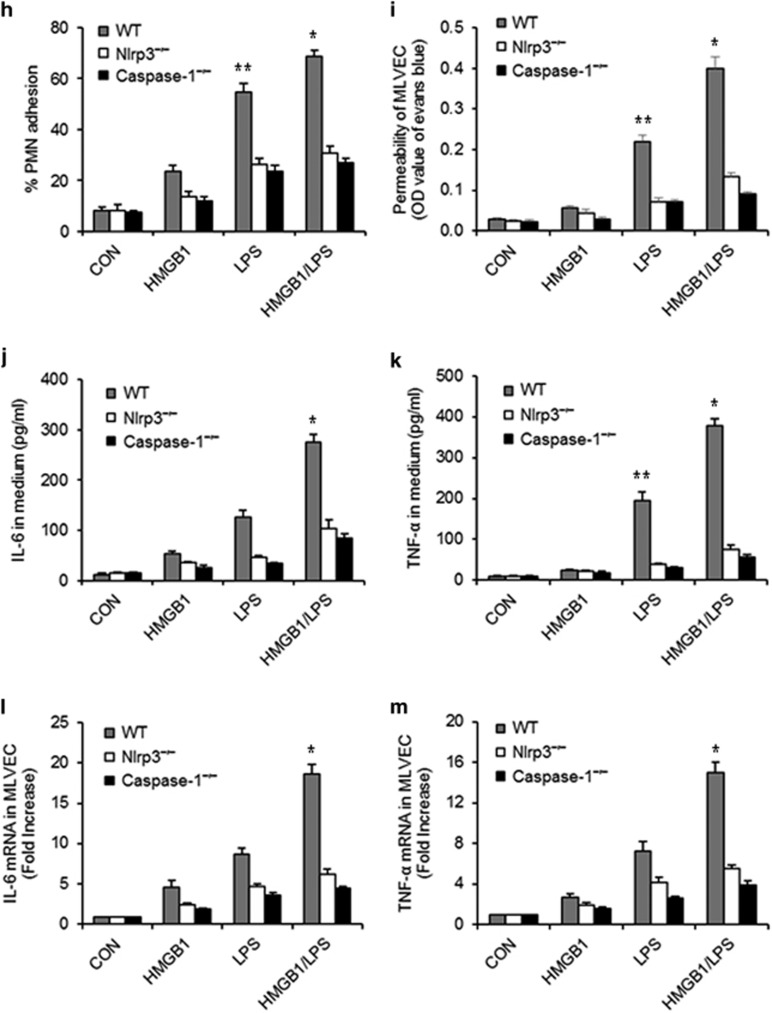
HS-primed EC pyroptosis enhances acute lung injury. (**a**–**g**) WT, RAGE^−/−^, and caspase-1^−/−^ mice were subjected to HS or sham operation (SM) followed by LPS or saline (SAL) i.t. at 2 h after HS. At 24 h after LPS i.t., lung histology was assessed with H&E staining (original magnification × 400) (**a**); lung tissue wet/dry ratio was measured (**b**); lung tissue MPO activity was measured using a murine MPO activity assay kit (**c**); protein concentration in BALF was measured by Lowry method (**d**); and IL-1*β*, IL-6, and TNF-*α* concentrations in BALF were determined by ELISA (**e**–**g**). (**h**–**k**) MLVEC isolated from WT, RAGE^−/−^, and caspase-1^−/−^ were sequentially treated with HMGB1 (0.5 *μ*g/ml) for 4 h and then with LPS (1 *μ*g/ml) for 24 h. PMN (1 × 10^5^ cells) isolated from WT circulating blood were then added onto the surface of the treated MLVEC and incubated for 30 min, and the percentage of adherent PMN was counted under microscope (**h**); Endothelial permeability was assessed by Evans blue-labeled BSA (**i**); and IL-6, and TNF-*α* in the cell culture medium were measured by ELISA (**j** and **k**). (**l** and **m**) MLVEC derived from WT mice, RAGE^−/−^ mice, and Caspase-1^−/−^ mice were treated with HMGB1 (0.5 *μ*g/ml) for 4 h and/or LPS (1 *μ*g/ml) for 24 h to induce pyroptosis in the MLVEC in the upper well of Transwell, followed by co-incubating with untreated WT MLVEC, which were in the bottom well of Transwell, for additional 6 h. IL-6 and TNF-*α* mRNA levels in the WT MLVEC in the bottom well were then measured by qRT-PCR. All images are representatives of five independent experiments, and graphs depict the value of mean and S.E.M. *P<0.05 compared with the groups labeled with no or different asterisk, **P<0.05 compared with the groups labeled with no or different asterisk, ^#^*P*<0.05 between the two groups

To determine the role of lung EC pyroptosis in acute lung injury, WT, Nlrp3^−/−^, and caspase-1^−/−^ mice were subjected to HS–LPS ‘two-hit' model, and lung tissue and bronchoalveolar lavage fluid (BALF) were recovered at 24 h after LPS. Lung histology showed that HS–LPS induced a large amount of polymorphonuclear neutrophil (PMN) infiltration in alveoli and interstitial edema in WT mice, and these changes were attenuated in RAGE^−/−^ or caspase-1^−/−^ mice, in which HS failed to induce augmented lung EC pyroptosis (Figure 6a). Genetic deletion of RAGE or caspase-1 also significantly diminished HS–LPS-induced increases in lung wet/dry ratio (Figure 6b), lung tissue myeloperoxidase (MPO) activity (Figure 6c), and BALF protein concentration (Figure 6d). Additionally, as shown in Figures 6e–g, HS–LPS markedly increased IL-1*β*, IL-6, and TNF-*α* levels in BALF collected from WT mice, whereas, either RAGE deficiency or caspase-1 deficiency significantly decreased the inflammatory cytokines release in response to HS–LPS.

In order to distinct the roles of EC pyroptosis in the lung inflammation from other cell populations, *in vitro* studies using MLVEC were performed. MLVEC isolated from WT, RAGE^−/−^, and caspase-1^−/−^ mice were sequentially treated with HMGB1 and LPS to induce EC pyroptosis. At 24 h after the LPS treatment, PMN-EC adhesion, EC permeability, and IL-6 and TNF-*α* concentration in the culture medium were measured. As shown in Figures 6h–k, in WT EC, HMGB1-LPS induced marked increases in PMN-EC adhesion and EC permeability, as well as IL-6 and TNF-*α* release; whereas, RAGE deficiency or caspase-1 deficiency reduced the changes.

Furthermore, we evaluated the influence of pyroptotic EC on normal EC. After induction of MLVEC pyroptosis using HMGB1-LPS sequential treatment for 24 h, the pyroptotic EC were co-cultured with normal MLVEC in Transwell for 6 h. As shown in Figures 6l and m, pyroptotic MLVEC induced significant increases in TNF-*α* and IL-6 mRNA expression in non-pyroptotic cells.

## Discussion

**Figure 7:**
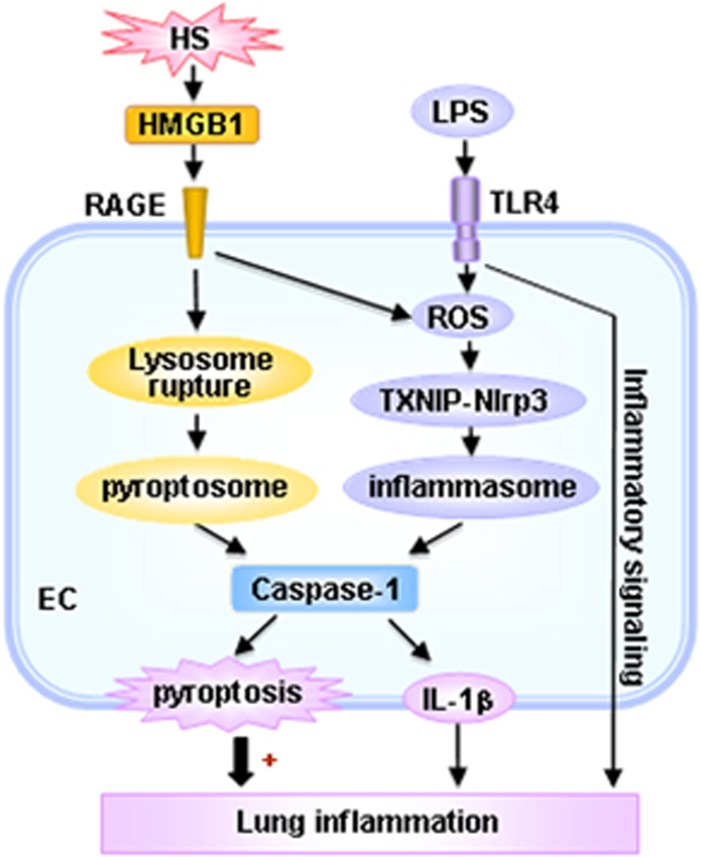
Hypothetic model for HS-primed lung EC pyroptosis and lung inflammation in response to LPS. LPS through TLR4 activates Nlrp3 inflammasome in MLVEC, and consequently induces caspase-1 activation. On the other aspect, HS induced release of HMGB1 through RAGE signaling initiates EC endocytosis of HMGB1, which in turn triggers CatB release from ruptured lysosomes followed by pyroptosome formation and caspase-1 activation. These HS-induced events enhance LPS-induced EC pyroptosis and subsequent exaggerated lung inflammation and injury

Pulmonary EC are the first cells of the lung to be altered in ALI/ARDS triggered by sepsis, trauma, and shock, and activated lung EC play an important role in the development of ALI.^4^ The role of lung EC pyroptosis in the pathogenesis of ALI and the underlying mechanism remain unclear. In this study, we showed that LPS through TLR4 activates Nlrp3 inflammasome in MLVEC, and consequently induces caspase-1 activation. From another aspect, HS induced release of HMGB1 through RAGE signaling initiates EC endocytosis of HMGB1, which in turn triggers CatB release from ruptured lysosomes followed by pyroptosome formation and caspase-1 activation. These HS-induced events enhance LPS-induced EC pyroptosis and subsequent exaggerated lung inflammation and injury (Figure 7).

HMGB1, as a DAMP molecule, contributes to pathobiology in both infectious and sterile inflammation. HMGB1 was originally described as a late mediator of sepsis.^25^ However, emerging evidences support that HMGB1 also serves as an early mediator in the setting of sterile inflammation, when it is released as a consequence of acute cellular stress or necrosis.^26, 27^ HMGB1 interacts with several receptors, including TLR2, TLR4, TLR9, and RAGE.^28, 29^ Administration of sub-lethal quantities of HMGB1 together with sub-lethal doses of LPS is synergistically toxic or lethal, indicating a role of HMGB1 in enhancing deleterious effect of LPS.^25^ However, how HMGB1 enhances inflammatory responses of host to LPS was unclear. In the current study, we revealed a novel role of HMGB1 in mediating HS-primed lung EC pyroptosis and subsequent augmented lung inflammation as well as underlying mechanism. We found that HS induced pyroptosome formation is required for enhanced lung EC pyroptosis in response to LPS, and the effect of HS on pyroptosome activation is mediated through RAGE-dynamin-CatB signaling pathway. This is evident by the observations that HS or HMGB1 significantly enhanced LPS-induced caspase-1 activation and lung EC pyroptosis, and neutralizing Ab against HMGB1 or RAGE deficiency effectively diminished the role of HS and HMGB1.

Pyroptosis is a form of cell death that features plasma membrane rupture and release of proinflammatory intracellular contents. Pyroptosis depends on caspase-1 activation, which is processed in inflammasome and pyroptosome.^30, 31, 32^

Inflammasome is a set of intracellular protein complexes that enable autocatalytic activation of caspase-1.^16^ The Nlrp3 inflammasome is currently the most fully characterized inflammasome and consists of the Nlrp3 scaffold, the ASC adaptor, and caspase-1. The Nlrp3 inflammasome can be activated upon exposure to pathogens, as well as a number of PAMPs, DAMPs, and environmental irritants.^33^ ROS have been suggested as an important activator of the inflammasome.^34^ In the present study, we demonstrated that LPS is able to activate Nlrp3 inflammasome as shown in Figures 3a and b. On the other aspect, HS-HMGB1 through RAGE enhances ROS production in lung EC in response to LPS. This is evident by the fact that RAGE deficiency markedly attenuated HMGB1-induced ROS production. We further elucidated that TXNIP may act as a sensor for changing levels of ROS, and consequently regulate Nlrp3 inflammasome activation. As shown in Figure 3, HMGB1 induced an increase in the association of TXNIP with Nlrp3 in response to LPS. The association of TXNIP and Nlrp3 was found to be essential for the subsequent caspase-1 activation, as silencing of TXNIP significantly attenuated HMGB1/LPS-induced Nlrp3 inflammasome assembly and caspase-1 activation.

Pyroptosome, also known as ASC foci, has been suggested to be the major machinery for pyroptosis induction.^24, 35^ Our recent study showed that HMGB1-induced alveolar macrophage pyroptosis requires pyroptosome assembly.^18^ In this study, we demonstrate that HMGB1 leads to pyroptosome assembly and subsequent caspase-1 activation in lung EC, and these consequences are crucial for augmenting LPS-induced EC pyroptosis. We showed that EC endocytosis of HMGB1, which is mediated through RAGE- and dynamin-dependent signaling, causes lysosome destabilization and CatB activation and release from the lysosome, and in turn, induces pyroptosome formation in the EC. As shown in the Results, either RAGE deficiency or the dynamin inhibitor dynasore effectively blocked HMGB1 internalization (Figures 4b and c), CatB activation (Figure 4g), and ASC foci formation (Figure 5a). Noteworthy, HMGB1 induced ASC foci formation was Nlrp3-independent, as Nlrp3 deficiency did not prevent the formation of ASC foci following HMGB1 stimulation. However, Nlrp3 deficiency prevented LPS-induced ASC assembly, suggesting an association of Nlrp3 and ASC in the formation of Nlrp3 inflammasome.

The caspase-1 knockout mice used in the study are believed to be linked with a caspase-11 deficiency. It has been reported that LPS from intracellular bacteria, through a TLR4-independent pathway, is able to directly bind to caspase-11 in mouse (caspase 4 or caspase 5 in human),^36, 37, 38, 39, 40^ in which guanylate-binding proteins facilitate the recognition of LPS from vacuolar bacteria.^41^ LPS binding results in the oligomerization and activation of caspase-11,^37^ which, in turn, cleaves gasdermin D to induce pyroptotic cell death.^42, 43, 44^ On the other aspect, active caspase-11 induces non-canonical activation of NLRP3 possibly by processing pannexin 1 and causing potassium efflux.^45^ As the method of LPS treatment used in the current study is unable to increase intracellular LPS concentration, and we have shown in the results that the LPS-induced lung EC pyroptosis is a TLR4-dependent event, the LPS-induced activation of caspase-11 and non-canonical Nlrp3 inflammasome does not seem to be a pathway that causes Nlrp3-dependent activation of caspase-1 in this study. Although the mechanism underlying caspase-1 induces cell pyroptosis has been unclear, recent report showed that caspase-1-cleaved gasdermin D protein can bind membrane lipids and form pores in the plasma membrane, and therefore, leading to lytic cell death.^46^

The lung EC have an important role in the development of lung inflammation.^47, 48, 49^ We investigated the influence of lung EC pyroptosis on the development of lung inflammation. We demonstrated that blocking EC pyroptosis by either genetic deletion of RAGE, which prevented HS/HMGB1-induced pyroptosome formation, or genetic deletion of caspase-1, which is required for cell pyroptosis, improved HS–LPS-induced lung inflammation and injury as shown in Figure 6. To exclude factors other than EC pyroptosis, in inducing inflammation, we investigated the direct impact of pyroptotic EC on normal EC using EC co-culture approaches. MLVEC pyroptosis was induced by the treatment of HMGB1+LPS for 24 h, and pyroptosis exhibited in about 46% of MLVEC as shown in Figure 2a. The pyroptotic MLVEC demonstrated effects on inducing TNF-*α* and IL-6 mRNA expression in non-pyroptotic cells. These data indicate a significant role for lung EC pyroptosis in promoting lung inflammation.

In summary, this study demonstrates a novel mechanism by which HS, through HMGB1-RAGE signaling, primes for lung EC pyroptosis in response to LPS, thereby, augmenting ALI. This study sheds light on the important role of EC pyroptosis in the development of post-HS inflammation.

## Materials and Methods

### Materials

Recombinant HMGB1 was purchased from R&D Systems (Minneapolis, MN, USA). Stimulating activity of recombinant HMGB1 was confirmed in mouse macrophages by assaying TNF release, with an ED50 of 3–12 *μ*g/ml. Neutralizing anti-HMGB1 IgY was purchased from SHINO-TEST Corporation (Kanagawa, Japan) and control nonimmune IgY was purchased from Fitzgerald (North Acton, MA, USA). All other chemicals were obtained from Sigma-Aldrich, except where noted.

### Mouse strains

All the mice used in the experiments were on a C57BL/6 background. WT C57BL/6 mice were purchased from the Jackson Laboratory (Bar Harbor, ME, USA). TLR4 knockout (TLR4^−/−^) mice, RAGE knockout (RAGE^−/−^) mice, Nlrp3 knockout (Nlrp3 ^−/−^) mice, and caspase-1 knockout (caspase-1^−/−^) mice were bred in Dr Timothy Billiar's laboratory at the University of Pittsburgh. All experimental protocols involving animals were performed in accordance with the recommendations in the Guide for the Care and Use of Laboratory Animals of the National Institutes of Health (Bethesda, MD, USA). All the animal experimental protocols were reviewed and approved by the Institutional Animal Care and Use Committee of VA Pittsburgh Healthcare System and University of Pittsburgh. All efforts were made to minimize suffering.

### Mouse model of HS and resuscitation

Mice were 12–14 weeks of age at the time of experiments and were maintained on standard rodent chow and water *ad libitum*. The mice were not fasted. Animals were anesthetized with 50 mg/kg ketamine and 5 mg/kg xylazine via i.p. administration. Femoral arteries were cannulated for monitoring of mean arterial pressure, blood withdrawal, and resuscitation. HS was initiated by blood withdrawal and reduction of the mean arterial pressure to 30 mm Hg within 20 min. Blood was collected into a 1-ml syringe and heparinized to prevent clotting. To exclude the effect of heparin on immune processes, equal amounts of heparin (10 U) were injected into sham animals through the cannulated femoral artery during the sham operation. After a hypotensive period of 2 h, animals were resuscitated by transfusion of the shed blood and Ringer's lactate in a volume equal to that of shed blood over a period of 20 min. The catheters were then removed, the femoral artery was ligated, and the incisions were closed. Sham animals underwent the same surgical procedures without hemorrhage and resuscitation. In some experiments, neutralizing IgY against HMGB1 (2 mg/kg BW) or nonimmune control IgY was injected i.p. into the mice 30 min before hemorrhage. At 2 h after resuscitation, LPS in a dose of 1 mg/kg body weight was injected i.t. into the mice (HS/LPS model). The animals remained anesthetized throughout the entire experimental period under the influence of ketamine and xylazine. At various time points after LPS injection (0–24 h), either BAL was performed and BALF was collected, or lung tissue was harvested for experimental analysis.

### MLVEC isolation and characterization

MLVEC were isolated using a previously described method^50^ that was modified in our laboratory as follows. Briefly, mice were anesthetized with 50 mg/kg ketamine and 5 mg/kg xylazine i.p. The chest cavity was opened, and the right ventricle was cannulated. PBS was infused to remove blood from lungs. Peripheral lung tissue dices in a size ~1 mm^3^ were prepared and cultured in a 60-mm culture dish in growth medium (MEM D-Val medium (Invitrogen Gibco, Grand Island, NY, USA) containing 2 mM glutamine, 10% fetal bovine serum (FBS), 5% human serum, 50 mg/ml penicillin/streptomycin, 5 mg/ml heparin, 1 mg/ml hydrocortisone, 80 mg/ml EC growth supplement from bovine brain, 5 mg/ml amphotericin, and 5 mg/ml mycoplasma removal agent) at 37 °C with 5% CO_2_ for 60 h. The adherent cells were continued in culture for 3 days after removal of the tissue dices, followed by purification using biotin-conjugated rat anti-mouse CD31 (PECAM-1) mAb and BD IMag streptavidin particles plus-DM, and the immunomagnetic separation system (BD Pharmingen, San Diego, CA, USA) following the manufacturer's instructions. The cells were allowed to grow for 3–4 days after purification. The cells were characterized by their cobblestone morphology, uptake of Dil-Ac-LDL (Biomedical Technologies, Stoughton, MA, USA), and staining for factor VIII-related Ag (Sigma Chemical, St. Louis, MO, USA). MLVEC were passaged three to five times before being used in experiments.

### Flow cytometry analysis of cell pyroptosis

Two-color flow cytometry was used to detect cell pyroptosis. MLVEC were incubated with Alexa Fluor 488-labeled caspase-1 FLICA at 37 °C for 1 h. After being fixed with 4% paraformaldehyde, cells were stained with TMR red-labeled In-Situ Cell Death Detection reagent (Roche Applied Science, Indianapolis, IN, USA) following the manufacturer's instructions. The cells were then analyzed by flow cytometry. Background and auto-fluorescence were determined by a control antibody with the same isotype staining. Acquisition was performed on 10 000 events using a FACScalibur cytometer (BD Biosciences, San Jose, CA, USA), CellQuestPro (BD Biosciences) and FlowJo-V10 software (Tree Star, Ashland, OR, USA). The double-stained cells were considered to be pyroptotic cells, and the rate of pyroptotic cell was calculated as (pyroptotic cells/total cells) × 100%.

### Coimmunoprecipitation and immunoblotting analysis

Mouse lung tissue or MLVEC were homogenized or lysed (1 × 10^6^ cells per ml) in lysis buffer (10 mM Tris (pH 7.4), 150 mM NaCl, 5 mM EDTA, 1% Triton X-100, 10 mM NaF, 1 mM Na3VO4, 10 *μ*g/ml leupeptin, 10 *μ*g/ml aprotinin, and 20 mM PMSF). The supernatants were quantified, and 600 *μ*g total protein for each sample was then immunoprecipitated with anti-ASC Ab (Santa Cruz Biotechnology, Santa Cruz, CA, USA) or anti-TXNIP Ab (MBL International, Ottawa, IL, USA). The immunoprecipitated proteins were separated on a 10% SDS-PAGE gel and then electroblotted onto polyvinylidene difluoride membrane and blocked for 1 h at room temperature with Odyssey Blocking Buffer (LI-COR Biotechnology, Lincoln, NE, USA). Nlrp3 was detected by probing the membranes with anti-Nlrp3 Ab (Santa Cruz Biotechnologies) at 1 : 500 dilution and detected with fluorescent secondary antibody (LI-COR Biotechnology) following the manufacturer's instructions. Blots were then stripped and reprobed with anti-ASC Ab or anti-TXNIP Ab and again detected with fluorescent secondary antibody (LI-COR Biotechnology). Caspase-1 cleavage in the lung tissue or MLVEC was measured by detecting its p10 fragment by western blot using rabbit polyclonal anti-mouse caspase-1 p10 (Santa Cruz Biotechnologies). TXNIP protein in MLVEC was detected by western blot using anti-TXNIP Ab.

### Immunofluorescence confocal microscopy

MLVEC were fixed with 4% paraformaldehyde for 20 min. After washing with PBS, the cells were permeabilized with 0.1% Triton X-100 in PBS for 10 min at room temperature, followed by blocking with 3% bovine serum albumin (BSA) in PBST (PBS with 0.1% Tween-20) for 2 h at room temperature to reduce nonspecific staining. The cells were then incubated with rabbit polyclonal anti-ASC Ab (Santa Cruz Biotechnology) at 4 °C overnight. After washing with PBS, the cells were incubated with Alexa Fluor 555-conjugated donkey anti-rabbit IgG (Abcam, Cambridge, MA, USA) for 1 h at room temperature. Hoechst 33258 (Sigma, St. Louis, MO, USA) was used to stain nuclei. The cells were then washed with PBS, followed by confocal microscopy.

### Cell staining and detection of lysosome rupture and CatB activation

Cell staining and detection of lysosome rupture and CatB activation were performed as previously described.^18^ MLVEC (5 × 10^4^ cells) were seeded onto a 35-mm Petri dish and grew with EC culture medium for 12 h at 37 °C. EGFP-tagged HMGB1 (HMGB1-EGFP) was added to the cells with a final concentration of 20 nmol/l. LysoTracker Red (75 nmol/l; Molecular Probes) was used together with the fluorescent recombinant HMGB1 for up to 12 h at 37 °C.

MLVEC cultured in 35 mm Petri dishes were treated with HMGB1 (20 nmol/l, 0.5 *μ*g/ml) and then stained with DQ Ovalbumin (10 *μ*g/ml, Molecular Probes) or Magic Red CatB assay reagent (10 *μ*g/ml, Immunochemistry Technologies, Bloomington, MN, USA) at 37 °C for 1 h for detection of lysosome rupture and CatB activity. The cells were then visualized by confocal microscopy.

### Measurement of IL-1*β*, IL-6, and TNF-*α*

IL-1*β*, IL-6, and TNF-*α* in BALF and cell culture media were measured using ELISA Ready-Set-Go kit for mouse IL-1*β*, IL-6 and TNF-*α* (eBioscience, San Diego, CA, USA), respectively, following the manufacturers' instructions.

### Measurement of myeloperoxidase activity

MPO activity in lung tissue was measured using Myeloperoxidase Activity Assay Kit (Abcam, Cambridge, MA, USA), according to the manufacturer's instruction.

### RNA extraction and quantitative real-time PCR

Total RNA was isolated from MLVEC by TRI Reagent (Molecular Research Center, Cincinnati, OH, USA) following manufacturer's instruction. Quantitative real-time PCR was done using a PrimerPCR SYBR Green Assay kit (Bio-Rad, Hercules, CA, USA) in a Bio-Rad iQ5 real-time PCR detection system (Bio-Rad Laboratories). The specific primers for mouse TNF-*α* and IL-6 were also purchased from Bio-Rad, and the assays were performed following the manufacturer's instruction. After amplification protocol was over, PCR product was subjected to melt curve analysis using Bio-Rad iQ5 software. Fold change in gene expression was calculated using the ΔΔC_T_ method ^51^ after normalizing to GAPDH and untreated MLVEC.

### Transfection of siRNA into MLVEC

TXNIP siRNA, control siRNA, and transfection kit were purchased from Santa Cruz Biotechnology Inc. MLVEC (2 × 10^5^ cells) were seeded in a six-well tissue-culture plate and incubated at 37 °C in a CO_2_ incubator until the cells were 80% confluent. The cells were then transfected with TXNIP siRNA or control siRNA using the siRNA transfection kit following the manufacturer's instructions. At 24–72 h after the transfection, TXNIP expression in the transfected cells was analyzed by western blot. Because we observed a confirmed knockdown of pyrin in the MLVEC at 48 h after siRNA transfection, we set this time point as time 0 for the experiments using HMGB1 and/or LPS treatment.

### *In vitro* PMN-MLVEC adhesion assay

The assay was performed as previously described.^52^ An immunomagnetic separation system (BD Biosciences Pharmingen)^53^ was used to isolate PMNs. Viability of the isolated PMNs was >95%, and PMN purity was >95% as assessed by trypan blue exclusion and Wright-Giemsa staining, respectively. MLVEC from WT, Nlrp3^−/−^, and RAGE^−/−^ mice were sequentially treated with HMGB1 (0.5 *μ*g/ml) for 4 h and then with LPS (1 *μ*g/ml) for 0–24 h. After treatment, the cells were incubated with PMNs derived from WT mice for 30 min. The non-adherent neutrophils were removed with gentle washing, and the percent of adherent PMN was calculated.

### *In vitro* MLVEC permeability assay

Permeability was quantified by spectrophotometric measurement of the flux of Evans blue-bound albumin across MLVEC monolayers using a two-compartment chamber model as previously described.^54, 55^ Briefly, MLVEC were plated (5 × 10^4^ cells per well) in 4-μm pore size and 12-mm diameter transwells for 3 days. The confluent monolayers were sequentially treated with HMGB1 (0.5 *μ*g/ml) for 4 h and LPS (1 *μ*g/ml) for 0–24 h. Inserts were washed with PBS, pH 7.4, before addition of 0.5 ml of 0.67 mg/ml Evans blue diluted in growth medium containing 4% BSA. Fresh growth medium was added to the lower chamber, and the medium in the upper chamber was replaced with Evans blue/BSA. After 10 min, the OD at 650 nm was measured in the lower chamber. Experiments were performed in triplicate and repeated five times.

### MLVEC–MLVEC coincubation

MLVEC–MLVEC coincubation was performed using Transwell plates (Corning Incorporated Life Sciences, Acton, MA, USA). MLVEC were sequentially treated with HMGB1 (0.5 *μ*g/ml) for 4 h and LPS (1 *μ*g/ml) for 24 h, and then transferred into the top well of Transwell in a concentration of 5 × 10^5^ cells per well. MLVEC in the bottom well were untreated. The co-cultures were then incubated for up to 6 h in DMEM containing 10% FBS.

### Data presentation and statistical analysis

The data are presented as mean±S.E.M. of the indicated number of experiments. SPSS 13.0 statistical analysis software (SPSS, Chicago, IL, USA) was used for statistical analysis. Differences among groups were determined by using one-way ANOVA or two-tailed Student's *t*-test and were considered statistically significant if the *P-*value was <0.05.

## Abbreviations

ALI: acute lung injury
ASC: apoptosis-associated speck-like protein containing a CARD domain
CatB: cathepsin B
EC: endothelial cell
HMGB1: high-mobility group box 1
HS: hemorrhagic shock
MLVEC: mouse lung vascular EC
MODS: multiple organ dysfunction syndrome
NLRs: NOD-like receptors
Nlrp3: NACHT, LRR and PYD domains containing protein 3
RAGE: receptor for advanced glycation end products
ROS: reactive oxygen species
SIRS: systemic inflammatory response syndrome
TXNIP: thioredoxin-interacting protein
